# Non-Invasive Prenatal Detection of Trisomy 13 Using a Single Nucleotide Polymorphism- and Informatics-Based Approach

**DOI:** 10.1371/journal.pone.0096677

**Published:** 2014-05-07

**Authors:** Megan P. Hall, Matthew Hill, Bernhard Zimmermann, Styrmir Sigurjonsson, Margaret Westemeyer, Jennifer Saucier, Zachary Demko, Matthew Rabinowitz

**Affiliations:** 1 Department of Research and Development, Natera Inc., San Carlos, California, United States of America; 2 Department of Genetic Counseling, Natera Inc., San Carlos, California, United States of America; Innsbruck Medical University, Austria

## Abstract

**Purpose:**

To determine how a single nucleotide polymorphism (SNP)- and informatics-based non-invasive prenatal aneuploidy test performs in detecting trisomy 13.

**Methods:**

Seventeen trisomy 13 and 51 age-matched euploid samples, randomly selected from a larger cohort, were analyzed. Cell-free DNA was isolated from maternal plasma, amplified in a single multiplex polymerase chain reaction assay that interrogated 19,488 SNPs covering chromosomes 13, 18, 21, X, and Y, and sequenced. Analysis and copy number identification involved a Bayesian-based maximum likelihood statistical method that generated chromosome- and sample-specific calculated accuracies.

**Results:**

Of the samples that passed a stringent DNA quality threshold (94.1%), the algorithm correctly identified 15/15 trisomy 13 and 49/49 euploid samples, for 320/320 correct copy number calls.

**Conclusions:**

This informatics- and SNP-based method accurately detects trisomy 13-affected fetuses non-invasively and with high calculated accuracy.

## Introduction

Trisomy 13 (Patau syndrome) results from one extra copy of chromosome 13, and is the third most common live-birth autosomal aneuploidy after trisomy 21 (Down syndrome) and trisomy 18 (Edward syndrome). The trisomy 13 live-birth incidence is between 1 in 10,000 and 1 in 20,000 [1], representing 33% of infants diagnosed prenatally via chorionic villus sampling (CVS) or amniocentesis [2]. The total prevalence for trisomy 13 live births, stillbirths, and elective terminations combined is approximately 1 in 6000 [3]–[6]. The trend towards delaying childbirth until more advanced maternal age has resulted in an increased incidence of all trisomic disorders, including trisomy 13 [7].

Complete, partial, and mosaic trisomy 13 have been documented [8]–[11]. Complete trisomy 13, involving a whole extra copy of the chromosome, results in severe, multi-systemic congenital anomalies, including central nervous defects, midline anomalies, eye and ear abnormalities, cardiac defects, orofacial defects, gastrointestinal and genitourinary abnormalities, limb defects, and developmental retardation [10], [11]. Most trisomic fetuses die in utero, and approximately 80% of trisomy 13-affected live births die within one month of birth [12]. Death in trisomy 13-affected fetuses is caused by multi-organ system failure, cardiopulmonary arrest, congenital heart disease, and pneumonia [6], [13]. However, there are a handful of cases where survival of up to 19 years has been observed [14]–[16]. Those who survive have serious intellectual and physical disabilities. Along with the maternal health risks associated with carrying any pregnancy, one small study specifically identified pre-eclampsia in mothers carrying trisomy 13 fetuses, but not in mothers carrying fetuses with other aneuploidies [17]. Early prenatal detection of trisomy 13 would allow parents time to come to terms with the diagnosis, receive counseling and prepare for the possible outcomes, and facilitate earlier decision-making.

Traditional prenatal diagnostic options include first and second trimester screening and/or diagnostic invasive procedures, like chorionic villus sampling (CVS) or amniocentesis. Non-invasive screening methods involving ultrasonography and biochemical analysis of maternal serum can be performed in the first trimester, but only detect at most 95% of trisomy 13 cases [18]. Additionally, these screening methods include a high rate of false positives and require follow-up invasive procedures for diagnosis. The timing of the screening protocols often delays diagnosis until the second trimester, prolonging maternal/parental emotional distress and delaying clinical decision-making. The high false-positive rate associated with traditional noninvasive screening methods, coupled with an approximate 1 in 500 risk of procedure-related pregnancy loss [19], results in a high number of unnecessary invasive procedures and thus lost pregnancies, many of which are euploid. Taken together, this emphasizes the need for a noninvasive method for detecting trisomy 13 early in pregnancy with detection rates and accuracy levels that mirror those of current invasive diagnostic methods.

The discovery of fetal cell-free DNA (cfDNA) in maternal blood suggested that such a test might be developed. However, fetal cfDNA is heavily diluted by maternal cfDNA, and the cfDNA is highly fragmented, complicating amplification and detection. Recent noninvasive methods circumvented these issues by amplifying isolated cfDNA using massively parallel shotgun sequencing (MPSS) and analyzing sequencing results using a quantitative counting approach [20]–[25]. These counting-based methods detect trisomic chromosomes by identifying chromosomes for which there is a higher relative abundance when compared to euploid reference chromosomes. Most MPSS-based counting methods non-specifically amplify cfDNA [20]–[22]. A more recent counting method termed digital analysis of selected regions (DANSR), uses targeted amplification and sequencing of cfDNA isolated from maternal plasma, thus reducing the number of required sequencing reads [23]–[26]. However, this method also relies on comparison with reference chromosomes to make copy-number calls. Both MPSS-based and DANSR methods accurately detect chromosomes 18 and 21, but are less reliable at chromosomes that suffer from amplification variation, including chromosome 13. Indeed, trisomy 13 detection rates in published studies describing commercially available methods range from 78.6% to 91.7% [20], [22], [26]. Correcting for amplification bias due to variation in the guanosine-cytosine levels has recently been shown to improve trisomy 13 detection rates [27], but this method is not commercially available. Thus, there is currently no clinically available noninvasive method that consistently and accurately detects trisomy 13.

Here, we present a cohort of 68 samples where trisomy 13 was accurately detected using targeted amplification and sequencing of single-nucleotide polymorphisms (SNPs) coupled with Next-generation Aneuploidy Test Using SNPs (NATUS) analysis.

## Materials and Methods

### Ethics Statement

Pregnant couples with an affected fetus or considered to be at high-risk for fetal aneuploidy (positive serum screen, ultrasound abnormalities, or maternal age of greater than 35 years) were enrolled at participating prenatal care centers under a protocol approved for each individual center (Western Institutional Review Board, Ethical and Independent Review Services, Einstein Institutional Review Board, Polish Mother’s Memorial Hospital Institutional Review Board, Bio Medical Research Institute of America, Institutional Review Board of the Mt. Sinai School of Medicine), in accordance with applicable laws and regulations and the principles expressed in the Declaration of Helsinki and the Belmont Report, between March and December of 2012. Women were at least 18 years of age, had singleton pregnancies, and signed an informed consent. Samples were sent to a single reference laboratory (San Carlos, CA) for analysis. At this time, the NATUS algorithm has only been validated in singleton pregnancies [28], [29].

### Subjects and Sample Collection

This was a case-control study. The single sample with known fetal mosaicism was excluded from determination of sensitivity and specificity. Samples included in this analysis were selected from a cohort of >1,000 pregnancies that included 17 non-mosaic trisomy 13 samples (3 of which were previously reported) [28], [29]. All confirmed trisomy 13 cases were included in this study. Each sample from a woman carrying an aneuploid fetus was matched, to within five days of gestation, with three independent, randomly selected samples from pregnant women carrying euploid fetuses. Copy number on all samples was verified through standard invasive diagnostic testing (amniocentesis or chorionic villus sampling [CVS]) or genetic testing of the cord blood, buccal, saliva, or products of conception.

### Sample Preparation and Measurement

Samples were prepared and amplified using 11,000-plex or 19,488-plex targeted polymerase chain reaction (PCR) in solution, and analyzed as described previously [28]–[30]. Probes of between 18 and 30 base pairs (bps) in length were designed to minimize primer-primer interactions and to generate amplicons of 50 to 65 bp in length [31]–[34]. Isolated cfDNA as well as maternal and paternal genomic DNA samples were pre-amplified for 15 cycles using 11,000 or 19,488 target-specific assays. Next, an aliquot was transferred to a second nested 15-cycle PCR before a third round of 12-cycle PCR where samples were prepared for sequencing by adding barcoded tags [28]. DNA integrity was measured via LabChip (Perkin Elmer, Waltham, MA). Targets included SNPs from chromosomes 13, 18, 21, X, and Y. Amplicons were sequenced using an Illumina GAIIx or HiSeq sequencer. SNPs targeted from the Y chromosome are in the homologous non-recombining regions of the X and Y chromosomes where the loci are common to the two chromosomes.

### NATUS Methodology and Data Analysis

Sequence alignment to the genome was performed using a proprietary algorithm adapted from the Novoalign (Novocraft, Selangor, Malaysia) commercial software package. A chromosome copy number classification algorithm called Next-generation Aneuploidy Test Using SNPs (NATUS) was implemented in MATLAB (MathWorks, Natick, MA, USA) [32]–[34]. The NATUS algorithm leverages an advanced version of the proprietary Parental Support statistical algorithm, which was previously described (and was used to analyze two trisomy 13 samples included here) [28]–[30], [32]–[34]. Briefly, the NATUS algorithm considers parental genotypic information, crossover frequency data [35], and linkage disequilibrium to predict possible fetal euploid and aneuploid genotypes. The algorithm then predicts what the sequencing data would be expected to look like for each of these hypothetical genotypes at various fetal cfDNA fractions; by comparing to the actual sequencing data, the algorithm identifies the combination of fetal genotype and fetal fraction that represents the maximum likelihood, and calls this as fetal copy number and fetal fraction. NATUS calculates a sample-specific accuracy for each interrogated chromosome. The calculated accuracy represents the likelihood that the copy number call is correct, and is expressed as a proportion of the maximum value of 1, which equates to a calculated accuracy of 100% [28]. To ensure that data is of sufficient clarity to result in a high-confidence result, NATUS considers numerous quality control metrics, which samples must pass for results to be reported [28]–[30]. Samples with <4.0% fetal fraction, <1,500 genome equivalents (where 1 genome equivalent is equal to the amount of DNA contained within a single cell), contamination >0.2% (where contamination was identified by the algorithm as additional genotypes present in a sample that do not match the parental or predicted fetal genotypes), or a signal-to-noise ratio or model fit that falls below previously-determined thresholds [28], [29], [32]–[34], were reported as a no-call. Although all samples that were part of this cohort were euploid or trisomy 13, the algorithm made copy number calls at five chromosomes (13, 18, 21, X and Y). Performance of the NATUS algorithm at chromosome 13 was compared to performance at chromosomes 18 and 21 for the 411 samples with karyotype confirmation via amniocentesis, CVS, or analysis of born child saliva.

## Results

**Figure 1:**
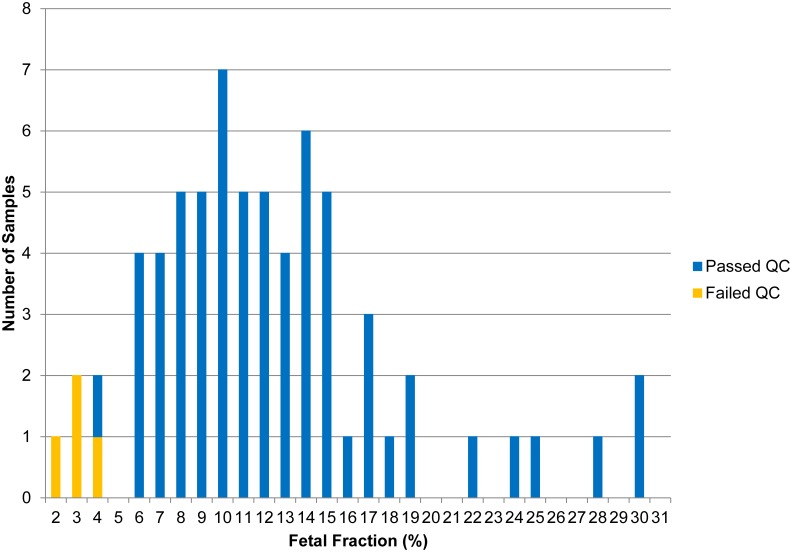
Histogram of samples stratified by fetal fraction.

The median gestational age (GA) was 16.0 weeks for euploid and aneuploid samples (overall range: 12.1–22.7 weeks). The mean measured fetal fraction for all samples was 12.1% (median: 11.1%, range: 2.2–30.4%). Four (5.9%) samples did not pass stringent quality control metrics; three (2 trisomy 13, 1 unaffected) were identified with fetal fractions of below the 1^st^ percentile (2.7%) when compared to euploid samples (see Discussion); the 4^th^ sample (unaffected) had a fetal fraction of above the 1^st^ percentile but below the threshold for making a copy number call. Figure 1 depicts the distribution of samples that passed quality control as a function of fetal fraction.

To determine whether chromosome 13 showed systematic differences from chromosomes 21 and 18 that could result in differing accuracies, the model fit quality for chromosomes 13, 18, and 21 was compared for 411 samples (as described in the Materials and Methods). For each sample, the NATUS algorithm calculated model fit on a per-SNP basis for each chromosome. The fit for all SNPs was then combined to calculate an aggregate fit for the various hypotheses under consideration (monosomy, disomy, trisomy). This aggregate model fit showed no observable difference between the three chromosomes, indicating that NATUS performs the same at chromosome 13 as it does at chromosomes 18 and 21. This consistency in performance is reflected in the accuracy of the copy number calls for the cohort reported here. As in previous studies, the single mosaic trisomy 13 case was excluded from calculations of sensitivity and specificity [22], [36]–[38]. For trisomy 13, sensitivity was 100% (15/15, CI: 78.2–100%) and specificity was 100% (49/49, CI: 92.8–100%). All samples were concurrently analyzed for trisomy 18, trisomy 21, and monosomy X, and specificity was 100% (64/64, CI: 94.4–100%). This resulted in a total of 320/320 correct copy number calls. The overall average calculated accuracy across interrogated chromosomes was >99%. Table 1 reports gestational ages, fetal fractions, NATUS-called karyotypes, average confidences, and confirmed karyotypes for sample passing quality control.

**Table 1:** List of 64 samples that passed quality control, with gestational age, fetal fraction, NATUS-generated copy number result, calculated accuracy, and confirmed karyotype.

GA (weeks)	Fetal Fraction (%)	NATUS-calledKaryotype	AverageConfidence^a^	Confirmed Karyotype^b^
12.1	11.4	46,XX	1.00	46,XX
12.1	10.3	46,XX	1.00	46,XX
12.7	14.9	46,XX	1.00	46,XX
12.7	6.7	46,XX	1.00	46,XX
12.7	8.7	46,XX	1.00	46,XX
15.6	15.2	46,XX	1.00	46,XX
15.6	12.7	46,XX	1.00	46,XX
18.0	8.3	46,XX	1.00	46,XX
16.0	12.5	46,XX	1.00	46,XX
16.0	10.4	46,XX	1.00	46,XX
16.0	15.5	46,XX	1.00	46,XX
15.9	15.2	46,XX	1.00	46,XX
18.1	13.8	46,XX	1.00	46,XX
18.9	9.7	46,XX	1.00	46,XX
13.1	11.7	46,XX	1.00	46,XX
13.1	5.9	46,XX	1.00	46,XX
12.4	6.4	46,XX	1.00	46,XX
16.4	7.1	46,XX	1.00	46,XX
16.4	28.1	46,XX	1.00	46,XX
21.1	7.6	46,XX	1.00	46,XX
23.1	21.9	46,XX	1.00	46,XX
12.1	10.8	46,XY	1.00	46,XY
12.7	13.2	46,XY	1.00	46,XY
12.7	9.9	46,XY	1.00	46,XY
13.6	12.2	46,XY	1.00	46,XY
13.6	16.6	46,XY	1.00	46,XY
13.6	14.0	46,XY	1.00	46,XY
13.6	8.7	46,XY	1.00	46,XY
15.6	9.9	46,XY	1.00	46,XY
18.0	13.9	46,XY	1.00	46,XY
18.0	6.4	46,XY	1.00	46,XY
18.1	10.5	46,XY	1.00	46,XY
15.9	14.1	46,XY	1.00	46,XY
15.9	13.4	46,XY	1.00	46,XY
18.9	30.4	46,XY	1.00	46,XY
18.9	14.9	46,XY	1.00	46,XY
21.4	14.0	46,XY	1.00	46,XY
21.4	17.8	46,XY	1.00	46,XY
21.9	23.5	46,XY	1.00	46,XY
21.1	12.0	46,XY	1.00	46,XY
21.1	16.7	46,XY	1.00	46,XY
21.1	7.8	46,XY	1.00	46,XY
22.6	10.8	46,XY	1.00	46,XY
22.4	17.0	46,XY	1.00	46,XY
22.7	9.7	46,XY	1.00	46,XY
13.1	15.3	46,XY	1.00	46,XY
12.4	9.9	46,XY	1.00	46,XY
12.4	9.0	46,XY	1.00	46,XY
16.4	18.9	46,XY	1.00	46,XY
18.0	24.7	47,XX,+13	1.00	47,XX,+13
15.9	12.0	47,XX,+13	1.00	47,XX,+13
21.6	19.2	47,XX,+13	1.00	47,XX,+13
13.1	4.3	47,XX,+13	0.92	47,XX,+13
13.6	5.5	47,XY,+13	1.00	47,XY,+13
15.6	8.5	47,XY,+13	1.00	47,XY,+13
16.0	7.1	47,XY,+13	1.00	47,XY,+13
18.9	7.7	47,XY,+13	1.00	47,XY,+13
22.6	10.8	47,XY,+13	1.00	47,XY,+13
21.6	11.6	47,XY,+13	1.00	47,XY,+13
18.1	14.2	47,XY,+13	1.00	47,XY,+13
22.0	30.0	47,XY,+13	1.00	47,XY,+13
12.1	9.1	47,XY,+13	1.00	47,XY,+13
12.7	7.5	47,XY,+13	1.00	Trisomy 13, no sex chromosome anomalies^b^
12.4	6.7	47,XY,+13	1.00	Trisomy 13, no sex chromosome anomalies^b^

For each trisomy 13 case, three confirmed euploid control cases with matching gestational ages (within five days) were blindly selected from a large collection of control cases.

a Average calculated accuracy [28]–[30] across chromosomes 13, 18, 21, and X.

b Karyotype was confirmed by standard invasive diagnostic testing or genetic testing of the cord blood, buccal, saliva, or products of conception. For two samples, confirmed fetal sex chromosome copy number was reported (from a larger, externally blinded cohort) as presence or absence of sex chromosome anomalies (instead of as “XX” or “XY”); NATUS-called karyotypes were identified as correct upon unblinding.

**Figure 2:**
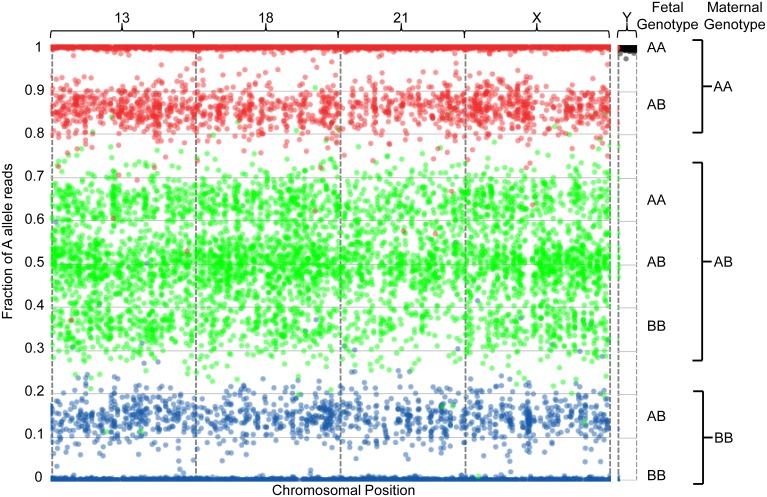
Graphical representation of sequencing data from one euploid sample with a 28.1% fetal fraction. SNPs are assumed to be binary (the algorithm ignores other minor alleles) and are indicated as A and B for simplicity. For each plot, the number of A allele reads is plotted as a fraction of the total allele reads (y-axis) against the linear position of each of several thousand interrogated SNPs on the chromosomes-of-interest (x-axis). The x-axis represents the linear position of each SNP along the indicated chromosome. Interrogated chromosomes are indicated above the plot. Each spot represents a single SNP, where the precise position along the y-axis represents the additive contribution of maternal and fetal cfDNA to the fraction of A allele reads and is thus a function of the sum of fetal and maternal allele reads for that locus as well as of fetal fraction. The contribution of reads from fetal alleles results in distribution of the spots into distinct clusters that can be used to infer chromosomal copy number. Fetal and maternal genotypes at individual SNPs are indicated to the right of the plots. To more easily visualize the maternal and fetal contributions, spots are color-coded according to maternal genotype: SNPs for which the mother is homozygous for the A allele (AA) are indicated in red, those for which the mother is homozygous for the B allele (BB) are indicated in blue, and those for which the mother is heterozygous (AB) are indicated in green. All clusters that are not tightly associated with the limits of the plots are useful for inferring ploidy, as described in the Results section.

**Figure 3:**
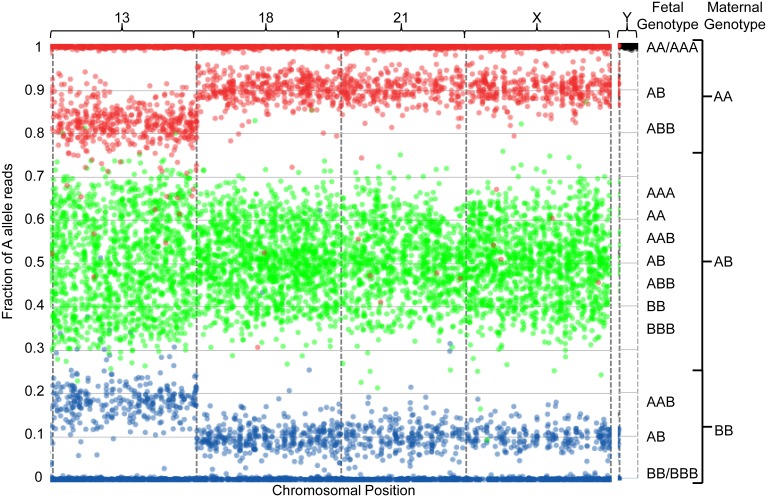
Graphical representation of sequencing data from one paternally-inherited trisomy 13 sample with a 19.2% fetal fraction. The plot is described as in Figure 2 and in the Results section.

**Figure 4:**
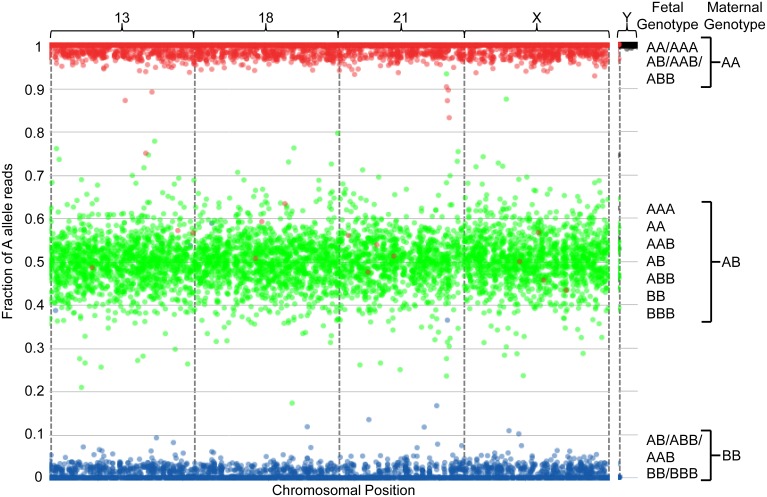
Graphical representation of sequencing data from one maternally-inherited trisomy 13 sample with a 4.2% fetal fraction. The plot is described as in Figure 2 and in the Results section.

The sequencing data can be visualized graphically, as in Figures 2–4. It is important to note that this is not how the algorithm makes copy number calls, but is meant as a straightforward method for visualizing results generated by the algorithm. Figure 2 depicts the data obtained from one 46,XX sample with a fetal fraction of 28.1%. The presence of three green clusters in the center of the plot (centered around 0.64, 0.5, and 0.36 as a function of fetal fraction), as well as two red peripheral clusters (one external and centered around 1, and one internal and centered around 0.86) and two blue peripheral clusters (one external and centered around 0, and one internal and centered around 0.14), are a hallmark pattern indicating the presence of two chromosomes. Thus, this fetus has two copies of chromosomes 13, 18, 21, and X. Together with the absence of reads from the Y chromosome (where spots are tightly associated with the upper limit of the plot, as described previously^30^), this indicates a female euploid fetal genotype.

The graphical representation in Figure 3 depicts a sample identified as having a 47,XX,+13 fetal genotype with a fetal fraction of 19.2%. The hallmark “two chromosome” clustering pattern is observed for chromosomes 18, 21, and X. In this case, the central green clusters have condensed towards the center of the plot (centered around 0.6, 0.5, and 0.4), and the peripheral red and blue clusters have regressed towards the upper and lower limits of the plot (centered around 0.9 and 0.1, respectively); this is due to the slightly lower fetal fraction when compared to Figure 2. For chromosome 13, the center green clusters for which the maternal genotype is heterozygous are distributed in four clusters (centered around 0.63, 0.54, 0.46, and 0.37); this pattern indicates fetal genotypes of AAA, AAB, ABB, and BBB, respectively. Additionally, the internal peripheral red and blue clusters have shifted towards the center of the plot (centered around 0.82 and 0.18, respectively). This hallmark pattern indicates a paternal mitotic error resulting in the paternal contribution of two alleles and thus the presence of three chromosomes in the fetus. Together with the absence of Y-chromosome reads, this indicates a female trisomy 13 fetal genotype.

The graphical representation in Figure 4 depicts a sample identified as having a 47,XX,+13 fetal genotype with a fetal fraction of 4.3%. At fetal fractions of below approximately 20%, the hallmark patterns that indicate disomy, trisomy or monosomy are not readily discernible by eye. However, the algorithm is able to make high-confidence copy number calls at as low as 3.8% fetal fraction. Due to the decreased fetal fraction in Figure 4, the peripheral red and blue clusters have regressed towards the plot’s upper and lower limits, respectively, and the central green clusters have condensed towards the plot’s center. This highlights the advantage of incorporating genotyping information; the calculated fit for each SNP permits a high-confidence aggregate model fit to ensure detection of fetal aneuploidy with high accuracy across a range of fetal fractions.

## Discussion

Here, we describe the application of a SNP- and informatics-based method for the detection of trisomy 13. The NATUS algorithm specifically amplifies and sequences SNPs from cfDNA isolated from maternal blood, targeting either 11,000 or 19,488 individual loci and using Bayesian-based Maximum Likelihood informatics analysis to detect trisomy [28]–[30]. The Parental Support method was originally developed to measure chromosome copy number at all 24 chromosomes in a single cell.^37^ The ability of the Parental Support method to call copy number at low DNA levels facilitated its adaptation as a method to noninvasively detect fetal aneuploidy, using the advanced NATUS version of the algorithm [28]–[30]. Because of the small number of trisomy 13 cases in previous reports [28], [29], trisomy 13 sensitivity has not previously been evaluated. Here, the performance of this SNP-based NIPT was determined using a larger cohort of trisomy 13 cases, thus allowing trisomy 13 sensitivity to be estimated. Significantly, trisomy 13 sensitivity was not found to be significantly lower than that reported for trisomy 18 or trisomy 21 [28], [29].

Reports indicate that between 0% and 60% of parents continue a pregnancy known to be affected with trisomy 13. [2] However, neonatal treatment is controversial due to high infant mortality rates, even though approximately 50% of trisomy 13 infants live beyond one week and up to 10% live beyond one year [39], [40]. An earlier diagnosis would offer mothers/parents the full range of pregnancy options.

There are currently no reliable noninvasive methods for detecting trisomy 13 that are commercially available. Counting methods all require reference chromosomes, which is problematic in that significant amplification variation or undetected reference chromosome aneuploidy may result in missed calls. Indeed, amplification variation, thought to be due to low GC content, is well-established as problematic for detecting aneuploidy at chromosome 13 [41]–[44], and as a result the published trisomy 13 detection rates of 78.6% to 91.7% are well below the diagnostic accuracy of invasive methods [22], [23]. This renders questionable the clinical utility of these methods as an eventual replacement for or adjunct to invasive methods in trisomy 13 detection. Improved accuracy at detecting both trisomy 13 and trisomy 18 was recently reported using a non-repeat-masked reference genome, bioinformatically correcting for GC content bias [27]. Although this modified method accurately detected 100% of trisomy 13 fetuses and 91.9% of trisomy 18 fetuses, the method still utilizes a non-targeted MPSS-based counting approach and fails to address fetal fraction. Thus, the method is still subject to issues with unnecessary reads, undetected reference chromosome aneuploidy, and is likely to be less accurate at low fetal fractions. By specifically interrogating polymorphic loci, the method presented here obviates issues with chromosome-to-chromosome amplification variation that result in decreased reliability at these chromosomes in counting methods. Specifically, amplification of polymorphic loci is inherently more consistent than amplification of other chromosomal regions, because the two alleles at a polymorphic locus by definition only differ by a single nucleotide. Thus, although all methods identify trisomy 18 and trisomy 21 with high accuracy, the NATUS method is the only commercially available method that detects all five chromosomes (13, 18, 21, X, and Y) with equally high accuracy. Although Liao *et al.* utilized targeted amplification and detection of SNPs, and SNP-based methods are expected to accurately call ploidy at all 24 chromosomes, the method is not commercially available, still relies on reference chromosomes, and only interrogated chromosome 21 [45].

As fetal cfDNA is believed to be placental in origin, the ability of NIPT to accurately report the fetal chromosome copy number is complicated by mosaicism. Confined placental mosaicism (CPM), typically associated with a euploid fetus, may result in cfDNA that is not representative of the actual fetal genetic state. Thus, mosaicism is considered to be an inherent limitation of all NIPTs, and historically, published NIPT studies have excluded mosaic cases when determining assay performance [20]–[26], [36], [37]. The cohort presented also excluded one case of fetal trisomy 13 mosaicism; for this case, the algorithm failed to return a result because of low fetal fraction, so the capacity for this SNP-based NIPT to detect mosaic cases cannot be evaluated.

The method presented here utilizes NATUS analysis, an advanced informatics approach to analyzing sequencing data. Whereas previous methods rely on read counts or intensity analysis, this method analyzes the prevalence and identity of individual SNPs, and includes parental genotypes coupled with crossover frequency information from the HapMap database [35]. Specifically, the method formulates billions of possible fetal euploid and aneuploid genotypes based on parental genotype information and crossover frequency data. It then predicts the expected allele distributions for each of the hypothetical genotypes at various fetal cfDNA fractions. By comparing the actual sequencing data to the predicted plasma DNA profiles and fetal cfDNA fractions using Bayesian statistics, the NATUS algorithm identifies both the fetal ploidy state and fetal fraction with the maximum likelihood. Significantly, this method performs a sophisticated DNA quality evaluation that takes into account fetal fraction, parental genotypes, noise parameters, sequence reads parameters, and a number of internal controls.

The method also calculates a sample-specific accuracy, which can be converted to a personalized risk score. Importantly, these sample-specific calculated accuracies are expected to offer more reliable results at low fetal fractions, where MPSS-based and DANSR methods may falter. Given that fetal fractions tend to be lower at early gestational ages and in trisomy 13 pregnancies [46], [47], this suggests that this method will either provide accurate ploidy calls at low fetal fraction, or will identify those samples with questionable DNA quality. This is distinct from the currently available methods, which all utilize cohort-based accuracies like z-score, and which are unable to detect individual samples with low accuracy in a larger cohort with a high overall accuracy. Because the NATUS method flags questionable samples, it is thus expected to reduce the number of missed calls.

One recent study reported that samples identified with a gestational age- and maternal weight-corrected fetal fraction of below the 0.5^th^ percentile when compared to euploid pregnanices were more likely to be affected with triploidy [48]. This increased risk was likely explained by the fact that digynic triploidies are associated with decreased fetal fractions due to small placental mass. As trisomy 13 pregnancies have been reported with below-average fetal fractions [47], [49], similar effects might be expected in this cohort. However, in this cohort maternal weight was not available. Despite this, 3 of the 4 no-calls were found to have uncorrected fetal fractions of below the 1^st^ percentile (2.7%) when compared to euploid pregnancies; 2 of these were trisomy 13-affected samples. It is thus possible that the abnormally low fetal fractions observed in trisomy 13 pregnancies in this cohort may also suggest an increased risk for aneuploidy.

In conclusion, this SNP-based, noninvasive method that employs NATUS analysis accurately detects fetal trisomy 13 from cfDNA isolated from maternal plasma. Given that this method employs highly efficient targeted sequencing, this method is expected to offer a clinically attractive method for accurate early detection of fetal trisomy 13.

## References

[1] Carey JC (2010) Trisomy 18 and Trisomy 13 Syndromes. In: Cassidy SB, Allanson JE, editors. Management of Genetic Syndromes. Hoboken, NJ: John Wiley & Sons, Inc.

[2] Lakovschek IC, Streubel B, Ulm B (2011) Natural outcome of trisomy 13, trisomy 18, and triploidy after prenatal diagnosis. American Journal of Medical Genetics, Part A 155: 2626–2633. 10.1002/ajmg.a.34284 21990236

[3] Crider KS, Olney RS, Cragan JD (2008) Trisomies 13 and 18: Population prevalences, characteristics, and prenatal diagnosis, metropolitan Atlanta, 1994–2003. American Journal of Medical Genetics, Part A 146A: 820–826. 18348276 10.1002/ajmg.a.32200

[4] Parker MJ, Budd JL, Draper ES, Young ID (2003) Trisomy 13 and trisomy 18 in a defined population: epidemiological, genetic and prenatal observations. Prenatal Diagnosis 23: 856–860. 14558033 10.1002/pd.707

[5] Forrester MB, Merz RD (2003) First-year mortality rates for selected birth defects, Hawaii, 1986–1999. American Journal of Medical Genetics, Part A 119A: 311–318. 12784299 10.1002/ajmg.a.20151

[6] Vendola C, Canfield M, Daiger SP, Gambello M, Hashmi SS, et al (2010) Survival of Texas infants born with trisomies 21, 18, and 13. American Journal of Medical Genetics, Part A 152A: 360–366. 20082470 10.1002/ajmg.a.33156

[7] Egan JF, Benn P, Borgida AF, Rodis JF, Campbell WA, et al (2000) Efficacy of screening for fetal Down syndrome in the United States from 1974 to 1997. Obstetrics and Gynecology 96: 979–985. 11084189 10.1016/s0029-7844(00)01044-9

[8] Buyse ML (1990) Birth Defects Encyclopedia. Cambridge, MA: Blackwell Scientific Publications.

[9] Griffith CB, Vance GH, Weaver DD (2009) Phenotypic variability in trisomy 13 mosaicism: two new patients and literature review. American Journal of Medical Genetics, Part A 149A: 1346. 19449431 10.1002/ajmg.a.32883

[10] Pont SJ, Robbins JM, Bird TM, Gibson JB, Cleves MA, et al (2006) Congenital malformations among liveborn infants with trisomies 18 and 13. American Journal of Medical Genetics, Part A 140: 1749–1756. 16835915 10.1002/ajmg.a.31382

[11] Patau K, Smith DW, Therman E, Inhorn SL, Wagner HP (1960) Multiple congenital anomaly caused by an extra autosome. Lancet 275: 790–793. 10.1016/s0140-6736(60)90676-0 14430807

[12] Hassold TJ, Jacobs PA (1984) Trisomy in man. Annual Review of Genetics 18: 69–97. 10.1146/annurev.ge.18.120184.000441 6241455

[13] Baty BJ, Blackburn BL, Carey JC (1994) Natural history of trisomy 18 and trisomy 13: I. Growth, physical assessment, medical histories, survival, and recurrence risk. American Journal of Medical Genetics, Part A 49: 175–188. 10.1002/ajmg.1320490204 8116665

[14] Mankinen CB, Sears JW (1976) Trisomy 13 in a female over 5 years of age. Journal of Medical Genetics 13: 157–161. 933114 10.1136/jmg.13.2.157 PMC1013378

[15] Cowen JM, Walker S, Harris F (1979) Trisomy 13 and extended survival. Journal of Medical Genetics 16: 155–157. 458834 10.1136/jmg.16.2.155 PMC1012743

[16] Redheendran R, Neu RL, Bannerman RM (1981) Long survival in trisomy-13-syndrome: 21 cases including prolonged survival in two patients 11 and 19 years old. American Journal of Medical Genetics, Part A 8: 167–172. 10.1002/ajmg.1320080207 7282771

[17] Boyd PA, Lindenbaum RH, Redman C (1987) Pre-eclampsia and trisomy 13: a possible association. Lancet 2: 425–427. 2887728 10.1016/s0140-6736(87)90960-3

[18] Spencer K, Nicolaides KH (2002) A first trimester trisomy 13/trisomy 18 risk algorithm combining fetal nuchal translucency thickness, maternal serum free beta-hCG and PAPP-A. Prenatal Diagnosis 22: 877–879. 12378569 10.1002/pd.420

[19] ACOG Practice Bulletin No. 88, December 2007. Invasive prenatal testing for aneuploidy. Obstet Gynecol 110: 1459–1467. 18055749 10.1097/01.AOG.0000291570.63450.44

[20] Palomaki GE, Deciu C, Kloza EM, Lambert-Messerlian GM, Haddow JE, et al (2012) DNA sequencing of maternal plasma reliably identifies trisomy 18 and trisomy 13 as well as Down syndrome: an international collaborative study. Genet Med 14: 296–305. 22281937 10.1038/gim.2011.73 PMC3938175

[21] Palomaki GE, Kloza EM, Lambert-Messerlian GM, Haddow JE, Neveux LM, et al (2011) DNA sequencing of maternal plasma to detect Down syndrome: an international clinical validation study. Genet Med 13: 913–920. 22005709 10.1097/GIM.0b013e3182368a0e

[22] Bianchi DW, Platt LD, Goldberg JD, Abuhamad AZ, Sehnert AJ, et al (2012) Genome-wide fetal aneuploidy detection by maternal plasma DNA sequencing. Obstet Gynecol 119: 890–901. 22362253 10.1097/AOG.0b013e31824fb482

[23] Sparks AB, Struble CA, Wang ET, Song K, Oliphant A (2012) Noninvasive prenatal detection and selective analysis of cell-free DNA obtained from maternal blood: evaluation for trisomy 21 and trisomy 18. Am J Obstet Gynecol 206 319: e311–319. 10.1016/j.ajog.2012.01.030 22464072

[24] Sparks AB, Wang ET, Struble CA, Barrett W, Stokowski R, et al (2012) Selective analysis of cell-free DNA in maternal blood for evaluation of fetal trisomy. Prenat Diagn 32: 3–9. 22223233 10.1002/pd.2922 PMC3500507

[25] Ashoor G, Syngelaki A, Wagner M, Birdir C, Nicolaides KH (2012) Chromosome-selective sequencing of maternal plasma cell-free DNA for first-trimester detection of trisomy 21 and trisomy 18. Am J Obstet Gynecol 206 322: e321–325. 10.1016/j.ajog.2012.01.029 22464073

[26] Ashoor G, Syngelaki A, Wang E, Struble C, Oliphant A, et al (2013) Trisomy 13 detection in the first trimester of pregnancy using a chromosome-selective cell-free DNA analysis method. Ultrasound Obstet Gynecol 41: 21–25. 22996646 10.1002/uog.12299

[27] Chen EZ, Chiu RW, Sun H, Akolekar R, Chan KC, et al (2011) Noninvasive prenatal diagnosis of fetal trisomy 18 and trisomy 13 by maternal plasma DNA sequencing. PLoS One 6: e21791. 21755002 10.1371/journal.pone.0021791 PMC3130771

[28] Zimmermann B, Hill M, Gemelos G, Demko Z, Banjevic M, et al (2012) Noninvasive prenatal aneuploidy testing of chromosomes 13, 18, 21, X, and Y, using targeted sequencing of polymorphic loci. Prenat Diagn 32: 1233–1241. 23108718 10.1002/pd.3993 PMC3548605

[29] Nicolaides KH, Syngelaki A, Gil M, Atanasova V, Markova D (2013) Validation of targeted sequencing of single-nucleotide polymorphisms for non-invasive prenatal detection of aneuploidy of chromosomes 13, 18, 21, X, and Y. Prenat Diagn. 33: 575–579. 10.1002/pd.4103 23613152

[30] Samango-Sprouse C, Banjevic M, Ryan A, Sigurjonsson S, Zimmermann B, et al (2013) SNP-based non-invasive prenatal testing detects sex chromosome aneuploidies with high accuracy. Prenat Diagn 33: 643–649. 23712453 10.1002/pd.4159 PMC3764608

[31] Zimmermann B, Hill M, Lacroute P, Dodd M (2013) Highly multiplex PCR methods and compositions. Unites States: Natera, Inc.

[32] Rabinowitz M, Gemelos G, Banjevic M, Ryan A, Demko Z, et al.. (2012) Methods for non-invasive prenatal ploidy calling. In: World Intellectual Property Organization IB, editor. G06F 19/22 (2011.01) G01N 33/48 (2006.01) ed. United States.

[33] Zimmermann B, Hill M, Lacroute P, Dodd M (2013) Highly multiplexed PCR methods and compositions. In: Office USP, editor. C12Q 1/68 (2006.01) ed. United States.

[34] Rabinowitz M, Banjevic M, Demko Z, Johnson DS (2013) Method for determining the number of copies of a chromosome in the genome of a target individual using genetic data from genetically related individuals. In: Office USP, editor. United States.

[35] Sherry ST, Ward M-H, Kholodov M, Baker J, Phan L, et al (2001) dbSNP: the NCBI database of genetic variation. Nucleic Acids Research 29: 308–311. 11125122 10.1093/nar/29.1.308 PMC29783

[36] Norton ME, Brar H, Weiss J, Karimi A, Laurent LC, et al (2012) Non-Invasive Chromosomal Evaluation (NICE) Study: results of a multicenter prospective cohort study for detection of fetal trisomy 21 and trisomy 18. Am J Obstet Gynecol 207 137: e131–138. 10.1016/j.ajog.2012.05.021 22742782

[37] Mazloom AR, Dzakula Z, Oeth P, Wang H, Jensen T, et al (2013) Noninvasive prenatal detection of sex chromosomal aneuploidies by sequencing circulating cell-free DNA from maternal plasma. Prenat Diagn 33: 591–597. 23592550 10.1002/pd.4127

[38] Nicolaides KH, Syngelaki A, Ashoor G, Birdir C, Touzet G (2012) Noninvasive prenatal testing for fetal trisomies in a routinely screened first-trimester population. Am J Obstet Gynecol 207 374: e371–376. 10.1016/j.ajog.2012.08.033 23107079

[39] Rasmussen SA, Wong LYC, Yang QY, May KM, Friedman JM (2003) Population-based analysis of mortality in trisomy 13 and trisomy 18. Pediatrics 111: 777–784. 12671111 10.1542/peds.111.4.777

[40] Tsukada K, Imataka G, Suzumura H, Arisaka O (2012) Better prognosis in newborns with trisomy 13 who received intensive treatments: a retrospective study of 16 patients. Cell Biochemistry and Biophysics 63: 191–198. 22487910 10.1007/s12013-012-9355-0 PMC3372784

[41] Fan HC, Blumenfeld YJ, Chitkara U, Hudgins L, Quake SR (2008) Noninvasive diagnosis of fetal aneuploidy by shotgun sequencing DNA from maternal blood. Proc Natl Acad Sci U S A 105: 16266–16271. 18838674 10.1073/pnas.0808319105 PMC2562413

[42] Chiu RW, Sun H, Akolekar R, Clouser C, Lee C, et al (2010) Maternal plasma DNA analysis with massively parallel sequencing by ligation for noninvasive prenatal diagnosis of trisomy 21. Clin Chem 56: 459–463. 20026875 10.1373/clinchem.2009.136507

[43] Alkan C, Kidd JM, Marques-Bonet T, Aksay G, Antonacci F, et al (2009) Personalized copy number and segmental duplication maps using next-generation sequencing. Nat Genet 41: 1061–1067. 19718026 10.1038/ng.437 PMC2875196

[44] Dohm JC, Lottaz C, Borodina T, Himmelbauer H (2008) Substantial biases in ultra-short read data sets from high-throughput DNA sequencing. Nucleic Acids Res 36: e105. 18660515 10.1093/nar/gkn425 PMC2532726

[45] Liao GJ, Chan KC, Jiang P, Sun H, Leung TY, et al (2012) Noninvasive prenatal diagnosis of fetal trisomy 21 by allelic ratio analysis using targeted massively parallel sequencing of maternal plasma DNA. PLoS One 7: e38154. 22666469 10.1371/journal.pone.0038154 PMC3362548

[46] Wegrzyn P, Faro C, Falcon O, Peralta CF, Nicolaides KH (2005) Placental volume measured by three-dimensional ultrasound at 11 to 13+6 weeks of gestation: relation to chromosomal defects. Ultrasound Obstet Gynecol 26: 28–32. 15937964 10.1002/uog.1923

[47] McFadden DE, Kalousek DK (2005) Two different phenotypes of fetuses with chromosomal triploidy: Correlation with parental origin of the extra haploid set. American Journal of Medical Genetics, Part A 38: 535–538. 10.1002/ajmg.1320380407 2063893

[48] Nicolaides K, Syngelaki A, Gil M, Quezada M, Zinevich Y (2013) Prenatal Detection of Fetal Triploidy from Cell-Free DNA Testing in Maternal Blood. Fetal Diagnosis and Therapy. 10.1159/000355655 24135152

[49] Choi H, Lau TK, Jiang FM, Chan MK, Zhang HY, et al (2013) Fetal aneuploidy screening by maternal plasma DNA sequencing: ‘false positive’ due to confined placental mosaicism. Prenat Diagn 33: 198–200. 23192749 10.1002/pd.4024

